# Membrane Emulsification Process as a Method for Obtaining Molecularly Imprinted Polymers

**DOI:** 10.3390/polym13162830

**Published:** 2021-08-23

**Authors:** Joanna Wolska, Nasim Jalilnejad Falizi

**Affiliations:** 1 Department of Process Engineering and Technology of Polymeric and Carbon Materials, Faculty of Chemistry, Wroclaw University of Science and Technology, Wybrzeze Wyspianskiego 27, 50-370 Wroclaw, Poland; 2 Chemical Engineering Department, Engineering Faculty, Ege University, Izmir 35100, Turkey; nasim.jalilnejad@gmail.com; 3 Biotechnology Division, Graduate School of Sciences, Ege University, Izmir 35100, Turkey

**Keywords:** bisphenol A, endocrine disruptors, membrane emulsification process, sorption, thermosensitive molecularly imprinted polymers

## Abstract

The membrane emulsification process (ME) using a metallic membrane was the first stage for preparing a spherical and monodisperse thermoresponsive molecularly imprinted polymer (TSMIP). In the second step of the preparation, after the ME process, the emulsion of monomers was then polymerized. Additionally, the synthesized TSMIP was fabricated using as a functional monomer N-isopropylacrylamide, which is thermosensitive. This special type of polymer was obtained for the recognition and determination of trace bisphenol A (BPA) in aqueous media. Two types of molecularly imprinted polymers (MIPs) were synthesized using amounts of BPA of 5 wt.% (MIP-2) and 7 wt.% (MIP-1) in the reaction mixtures. Additionally, a non-imprinted polymer (NIP) was also synthesized. Polymer MIP-2 showed thermocontrolled recognition for imprinted molecules and a higher binding capacity than its corresponding non-imprinted polymer and higher than other molecularly imprinted polymer (MIP-1). The best condition for the sorption process was at a temperature of 35 °C, that is, at a temperature close to the phase transition value for poly(N-isopropylacrylamide). Under these conditions, the highest levels of BPA removal from water were achieved and the highest adsorption capacity of MIP-2 was about 0.5 mmol g^−1^ (about 114.1 mg g^−1^) and was approximately 20% higher than for MIP-1 and NIP. It was also observed that during the kinetic studies, under these temperature conditions, MIP-2 sorbed BPA faster and with greater efficiency than its non-imprinted analogue.

## 1. Introduction

Membranes are an integral part of our everyday life and are used in many areas of industry (e.g., chemical, petrochemical, pharmaceutical, and food industries). However, new possibilities for using these materials in other areas are still being sought. One of them is a process using membranes to obtain single oil-in-water (o/w) or water-in-oil (w/o) emulsion systems as well as multiple emulsion systems, w/o/w, as well as o/w/o. This process is called membrane emulsification (ME). This technique has received increasing interest over the last 30 years as an alternative method to produce emulsions and particles and is a promising technique that allows the production of droplets of emulsion under controlled conditions with very low polydispersity [1,2]. Furthermore, the process requires a much lower energy input compared to traditional emulsification methods such as, e.g., rotating stirrer methods or homo-mixers [3,4].

In the membrane emulsification process, a membrane with a strictly defined pore size is used to obtain the emulsion. The dispersed phase is pressed through the pores of the membrane and then the droplets at their outlet are formatted. These droplets increase and after reaching the optimal size under the given conditions, they are “detached” by the continuous phase flowing near the surface of the membrane [4]. Their detachment occurs when the balance between the forces holding them at the outlet of the pores and the force detaching them from the membrane surface is disturbed. This technique, compared to conventional methods of emulsion preparation, requires using smaller forces to produce a dispersion, while the appropriate selection of each parameter (e.g., transmembrane pressure, membrane pore size, pores distribution, etc.) allows one to obtain the production of uniform droplets with controlled size [1,4,5].

During this study, the membrane emulsification method was adopted to receive the molecularly imprinted microspheres. After the ME process, the polymerization in microdroplets of monomers was carried out in the next stage of synthesis. This two-step method, compared to, e.g., conventional suspension polymerization, improves synthesis efficiency, is easy to carry out, and through an appropriate selection of process parameters, allows microspheres to be obtained with defined properties (the diameter of beads and their polydispersity) [6,7].

The materials synthesized during this study had imprints of bisphenol A (BPA), one of the compounds included in the so-called group of endocrine disrupting compounds (EDCs). In addition, the prepared polymers were synthesized with the use of one of the thermosensitive monomers, N-isopropylacrylamide (NIPAM), to create a kind of hybrid material, which would allow one to obtain a different effectiveness in removing BPA from the aqueous solutions. It was assumed that a change in the conformation of the polymer chains can change the shape and size of the cavities formed, and this phenomenon will result in differences in the removal of BPA and will facilitate the desorption process itself without the use of additional chemicals.

The most vital functions of the human body can be altered by endocrine disrupting compounds that interfere with the natural production, release, or elimination of hormones. These chemicals alter the performance of the endocrine system and can cause adverse health effects in humans and other mammals [8]. It has been observed, particularly during fetal development and early childhood, that exposure to low doses of EDCs (at the ppb level) can have serious effects on human life and reproduction, causing decreased mental capacity, genital abnormalities, and cancer [8]. There are varieties of molecules identified as EDCs, one of them being bisphenol A.

Bisphenol A, an industrial synthetic phenol, is widely used in the manufacture of many products such as engineering plastics, food cans [9], polycarbonate plastics, and epoxy resins. It should be emphasized that BPA is one of the most widely used synthetic compounds on the planet [10]. A worrying effect of bisphenol A is that it leaches out of food and beverage containers manufactured using BPA and contaminates the contents of the containers [10]. Furthermore, due to strong polarity and low volatility, aquatic environments are its main reservoirs. Therefore, the analysis and removal of trace BPA contamination from municipal wastewater, surface water, and groundwater is crucial. For the removal of bisphenol A, various methods have been used, e.g., adsorption, photodegradation, or biological treatment. Among these methods, adsorption is a superior and widely used method due to its mild operation conditions and low impact on the environment [11]. Unfortunately, traditional adsorbents appear to be powerless during use because of lack of selectivity. Thus, it is essential to develop novel adsorbents with high selectivity for the analysis and separation of trace levels of BPA in environment samples [12]. One type of sorbent is a material with special cavities in its structure, called molecularly imprinted polymer (MIP). MIPs are prepared using a template-assisted synthesis approach that generates selective recognition sites within a polymeric network and achieves receptor-like properties [13,14]. The size and shape of the imprints obtained are the same as those of the molecules used as a compound for their preparation. Molecularly imprinted polymers are promising sorbents for separation because they exhibit high affinity and selectivity similar to receptors for target molecules [13,15]. The latest reports from the literature [16,17,18] indicate that the molecular imprinting technique is an increasingly developing branch of material engineering that produces materials in various forms, for example, monoliths, membranes, or core-shell sorbents. They have been used in solid phase extraction [19,20,21], catalysis [22], or as chemical sensors [23].

Nowadays, a steadily growing number of scientific publications have focused on the development of sampling techniques and improving the final determination methods of ECDs, for example, BPA in environmental and biological samples [21,24] or plastic products [25]. Many research groups are looking for increasingly selective MIPs for the identification of BPA from various samples. An example of such materials can be MIPs containing magnetic particles [12,26], which are characterized not only by high selectivity toward BPA, but also due to the presence of magnetite, they are easier to separate and regenerate. Furthermore, the demand for fast biomedical, environmental, and food and beverage analysis is evolving very rapidly. Therefore, various new and advanced technologies are required to meet the new trends and requirements of analytical systems [27]. The use of MIPs as sensors is part of this trend. A new and widely developed direction for the use of MIP, especially in the form of nanospheres and microspheres, is its use in electrochemical sensors [28]. Increasingly, reports have appeared that conductive materials of this type are used to detect BPA in food, beverages, or water. In these structures, MIP is an identification element of electrochemical sensors for the detection of Bisphenol A [15,29,30,31,32].

Following the trend of searching for methods of identification and removal of BPA from water solutions, in the presented research, the membrane emulsification/suspension polymerization method will be explored for the fabrication of monodispersed microspherical molecularly imprinted polymers with BPA imprints. Moreover, these synthesized materials should be characterized with different BPA removal efficiency depending on the prevailing external temperature conditions.

## 2. Experimental

### 2.1. Reagents and Chemicals

N-isopropylacrylamide (NIPAM) and methyl methacrylate (MMA) purchased from Sigma-Aldrich (Poznan, Poland) were used as functional monomers. Ethylene glycol dimethacrylate (EGDMA) from Sigma-Aldrich (Poznan, Poland) was used as crosslinking agents, and azoisobutyronitrile (AIBN) from Aldrich (Poznan, Poland) was employed as an initiator. BPA from Sigma-Aldrich (Poznan, Poland) was used as a template and for the preparation of solutions for adsorption. Toluene (Avantor Performance Materials Ltd., Gliwice, Poland) was used as a porous agent. Methanol (Avantor Performance Materials Ltd., Gliwice, Poland) was used for the extraction of the prepared materials. Prior to use, MMA was purified by vacuum distillation. Other reagents were used as received. MilliQ water was used to obtain BPA solutions.

### 2.2. Apparatus

Commercial unit offered by Micropore Ltd. (Loughborough, UK) equipped with a metallic membrane having 20 µm of pore size was used for the membrane emulsification. The Quantachrome Autosorb 1-C analyzer (Anton Paar Ltd., Warsaw, Poland) was used to characterize the porous structure of the polymer beads. The sorption properties of the materials were determined using a UV–Vis Jasco V-630 spectrophotometer (Medson, Paczkowo, Poland). Scanning electron microscope micrographs of the prepared materials were obtained using a JSM 5800LV SEM microscope (JEOL Ltd., Warsaw, Poland). Attenuated Total Reflectance- Fourier-Transform Infrared Spectroscopy (ATR-FTIR) analysis was performed by means of the Jasco FT/IR-4700 spectrophotometer (Medson, Paczkowo, Poland).

### 2.3. Methods

#### 2.3.1. Preparation of Polymers

The sorbents shaped as the microbead particles were prepared from the monomers whose compositions are given in Table 1. For their preparation, a two-stage process was applied. In the first step, the membrane emulsification process was used, during which a mixture of NIPAM, MMA, EGDMA, AIBN, toluene, and BPA was passed through the metallic membrane and taken to the aqueous phase composed of 1 wt.% poly(vinyl alcohol) (MW 130 kDa) with 2 wt.% NaCl. In the second stage, the obtained emulsion was polymerized in a reactor. The membrane emulsification process was carried out in a commercial unit offered by Micropore Ltd. and is equipped with a metallic membrane having 20 µm of pores (for details, see [33]). Polymerization was carried out in a round-bottom flask equipped with a mechanical stirrer, at 60 °C for 48 h. After polymerization was complete, the mixture was cooled down, the microspheres obtained were filtered, washed with a large amount of water, dried and extracted with methanol in a Soxhlet apparatus for 24 h.

#### 2.3.2. Analysis of Materials

##### Scanning Electron Microscopy

Scanning electron microscopy (SEM) analysis was performed to obtain a more direct insight into the porous polymer structure using the JSM 5800LV model SEM device. Polymer beads were coated with gold to obtain a conductive surface. The acceleration potential was 15 kV. SEM micrographs of representative samples were taken with different (×5000, and ×20,000) magnifications.

##### Porous Structure Characterization

To characterize the porous structure of the obtained polymers, the nitrogen adsorption and desorption at the liquid nitrogen temperature were measured using the Quantachrome Autosorb 1-C analyzer. Before the analysis, all samples were outgassed at 25 °C for 12 h under high vacuum (3.75 × 10^−3^ Torr). The specific surface area (*S_BET_*) was calculated using the Brunauer–Emmett–Teller (BET) method. The total pore volume was derived from the amount of nitrogen gas adsorbed at a relative pressure close to unity, under the assumption that the pores were then filled with liquid adsorbate. The average pore diameter was obtained from the total pore volume and the BET surface area assuming cylindrical pore geometry [34]. The Barret–Joyner–Halenda (BJH) method with the Harkins–Jura equation and Faas correction was used to determine the pore size distribution (PSD) in the range of mesopores [35,36,37].

##### Nitrogen Content

The nitrogen content in the resins was determined using the Kjeldahl method after mineralization of the sample of a polymer (approximately 200 mg) in concentrated sulfuric acid containing copper sulfate and potassium sulfate [38].

##### Water Regain

The water regain (*W_H_*_2*O*_) (g H_2_O g^−1^) of the adsorbent was measured using the centrifugation method and was calculated from Equation (1):

(1) $$W_{H2O}=\frac{m_{s}-m_{d}}{m_{d}}$$

where *m_s_* (g) is the weight of the swollen polymer after centrifugation in a small column with a fritted-glass bottom and *m_d_* (g) is the weight of the polymer after drying at 105 °C for 24 h [39].

##### Characterization of Size and Polydispersity of Microspheres

The average size of the diameter microspheres and the SPAN number (the characteristic polydispersity index) were detected by means of Mastersizer X (Malvern Instruments GmbH, Germany). The polydispersity index was calculated from Equation (2) [40]:

(2) $$SPAN=\frac{d_{90}-d_{10}}{d_{50}}$$

where *d*_90_, *d*_50_, and *d*_10_ are the diameters of 90%, 50%, and 10% of the population of the spheres.

##### Fourier-Transform Infrared Spectroscopy (FT-IR)

To characterize the synthesized microbeads, the middle-infrared (4000–400 cm^−1^) spectra were collected by means of the Fourier-transform Jasco FT/IR-4700 model spectrometer. An investigated polymer sample (NIP or MIP) was placed on the diamond crystal of the attenuated total reflectance device. The spectral data were recorded at a resolution of 4 cm^−1^ by collecting 64 scans. The collected data were elaborated using Jasco Spectra Manager software.

#### 2.3.3. Evaluation of the Sorption Properties

##### Influence of Temperature

To study the influence of temperature on BPA uptake by all polymers prepared (NIP, MIP-1, and MIP-2), approximately 0.1 g of dry polymer particles and 50 mL of 0.5 mmol L^−1^ BPA solution at various temperatures (4, 25, 35 and 60 °C) were mixed for 24 h. After reaching equilibrium, the samples were filtrated, and the supernatant was analyzed for the concentration of BPA remained in the solution using a Jasco V–630 model spectrophotometer (λ_max_ = 276 nm). Next, the sorption capacity, *q_BPA_* (mmol g^−1^), defined as the amount of BPA adsorbed at the equilibrium, was calculated from Equation (3).

(3) $$q_{BPA}=\frac{\left(C_{0}-C_{e}\right)\cdot V}{m}$$

where *C_o_* and *C_e_* (mmol L^−1^) are the initial concentration of BPA and its concentration at equilibrium, respectively, *V* (L) is the volume of solution, and *m* (g) is the mass of dry polymer used [35].

Furthermore, the distribution coefficient (*K_BPA_*) was calculated as a ratio of the amount of BPA adsorbed on 1 g of polymer and the amount of bisphenol A at equilibrium in 1 mL of solution (Equation (4)) [41]:

(4) $$K_{BPA}=\frac{q_{BPA}\cdot \rho }{C_{e}}$$

where *ρ* (g L^−1^) is the density of BPA solution.

##### Adsorption Isotherms

To obtain adsorption isotherms, 0.1 g of dry adsorbent and 50 mL of solutions containing 0.2–2.0 mmol L^−1^ of BPA were shaken at 25 and 35 °C for 24 h. After reaching equilibrium, the adsorbents were separated from the solution by filtration and the concentration of BPA in the solution was determined using UV–Vis spectroscopy (λ_max_ = 276 nm). The adsorption capacity was calculated using Equation (1). Adsorption data were evaluated applying Langmuir, Freundlich, and Scatchard isotherms.

The Langmuir isotherm assumes that a monolayer of molecules is formed at the surface of the adsorbent. The linear form of the Langmuir equation can be given as follows (Equation (5)):

(5) $$\frac{1}{q_{e}}=\frac{1}{q_{m}b_{L}C_{e}}+\frac{1}{q_{m}}$$

where *q_e_* (mmol g^−1^) is the uptake at the equilibrium concentration, *q_m_* (mmol g^−1^) is the maximal uptake, *C_e_* (mmol L^−1^) is the equilibrium concentration, and *b_L_* (L mmol^−1^) is the constant related to the binding energy of the sorption system. Parameters *q_m_* and *b* were calculated from the slope and intercept of the linear plot of $\frac{1}{q_{e}}$ versus $\frac{1}{C_{e}}$ [42].

The next investigated model was the Freundlich isotherm. This model is assumed as a power function of the relationship between *q_e_* and *C_e_* and it is easily applicable when the experimental data are plotted in *log q_e_* versus *log C_e_* format (Equation (6)) [41,43]. The Freundlich isotherm is applicable to adsorption processes that occur on heterogeneous surfaces. This isotherm gives an expression that defines the surface heterogeneity and the exponential distribution of active sites and their energies. The linear form of the Freundlich isotherm is shown in Equation (6) [42,44]:

(6) $$logq_{e}=\frac{1}{n}\;logC_{e}+loga$$

The Freundlich equation (Equation (6)) served to calculate two fitting parameters *a* and $\frac{1}{n}$ that both yield a measure of physical binding. The *a* parameter is the constant related to the adsorption capacity. The $\frac{1}{n}$ parameter is known as the heterogeneity index. For homogeneous materials, $\frac{1}{n}$ would be equal to 1, the adsorption is linear, the adsorption sites are homogeneous in energy, and no interactions occur between the adsorbed compounds. On the other hand, when the values of the $\frac{1}{n}$ parameter approach zero, the heterogeneous character of the polymer increases [41,43,45]. The fitting of experimental data by the Freundlich isotherm allows to calculate the parameters a and $\frac{1}{n}$, which also helped to determine whether the selected model is appropriate.

The third model used was the Scatchard isotherm. This model is typically dedicated to MIPs. It allows the presence of two types of binding sites to be confirmed in the case of MIPs and confirms the heterogeneity of the surface of this type of polymers. The corresponding equation allows one to find the binding affinity (*K^S^_MIP_*) and the number of binding sites (*N*) [43]. In the Scatchard analysis, the experimental binding isotherm is plotted in $\frac{q_{e}}{C_{e}}$ versus *q_e_* format. In a homogeneous system that contains only one type of binding site, the Scatchard plot falls on one straight line with a slope equal to the negative value of binding affinity (*−K^S^*) and an *x*-intercept equal to the number of binding sites (*N*) (Equation (7)):

(7) $$\frac{q_{e}}{C_{e}}=K^{S}N-K^{S}q_{e}$$

For most MIPs, the Scatchard relationship shows curvature. It is considered as evidence for binding site heterogeneity. In that case, the analysis leads to modeling the isotherm by two separate straight lines. The limiting slope method yields two separate sets of binding parameters (*K^S^_MIP_*_1_*. N*_1_) and (*K^S^_MIP_*_2_*. N*_2_) for two classes of sites. The steeper line measures the high-affinity sites, and the gentle slope line measures the low-affinity sites [43]. For non-imprinted samples, NIPs, this trend cannot be observed.

##### Adsorption Kinetics

The adsorption kinetics of the selected polymers (MIP-2 and NIP) were studied at 35 °C. For this, 0.4 g dry polymer L^−1^ was contacted with a BPA solution (0.6 mmol L^−1^, 500 mL). A 3 mL of supernatant was taken out from the mixture at different time intervals to determine the concentration of BPA spectrophotometrically. Kinetic data were fitted to the diffusion models from the second Fick law, to find the rate (film diffusion (Equation (8)) or particle diffusion (Equation (9)) determining steps for sorbents [46,47]:

(8) $$k_{a}t=-\ln(1-(\frac{q_{t}}{q_{e}}))$$

where *q_t_* and *q_e_* (mmol g^−1^) represent the amount of adsorbed species at any time *t* (min) and at equilibrium time, respectively, and *k_a_* (min^−1^) represents the sorption rate constant. The sorption rate constant *k_a_* can be calculated from the plot of $-\ln(1-(\frac{q_{t}}{q_{e}}))$ versus time.

(9) $$k_{b}t=-\ln(1-(\frac{q_{t}}{q_{e}}{)}^{2})$$

where *k_b_* (min^−1^) is the sorption rate constant, *q_e_* and *q_t_* (mmol g^−1^) are the amount of adsorbed species at equilibrium and at time *t*. The sorption rate constant *k_b_* can be calculated from the plot of $-\ln(1-(\frac{q_{t}}{q_{e}}{)}^{2})$ versus time.

#### 2.3.4. Analysis of BPA Concentration

Jasco V-630 model spectrophotometer was used for the analysis of BPA concentrations (λ_max_ = 276 nm) in the solution. The uncertainty level for the concentration reading was estimated to 20–30 μmol L^−1^ level [41].

## 3. Results and Discussion

### 3.1. Preparation of Polymers and Characterization of Materials

**Figure 1:**
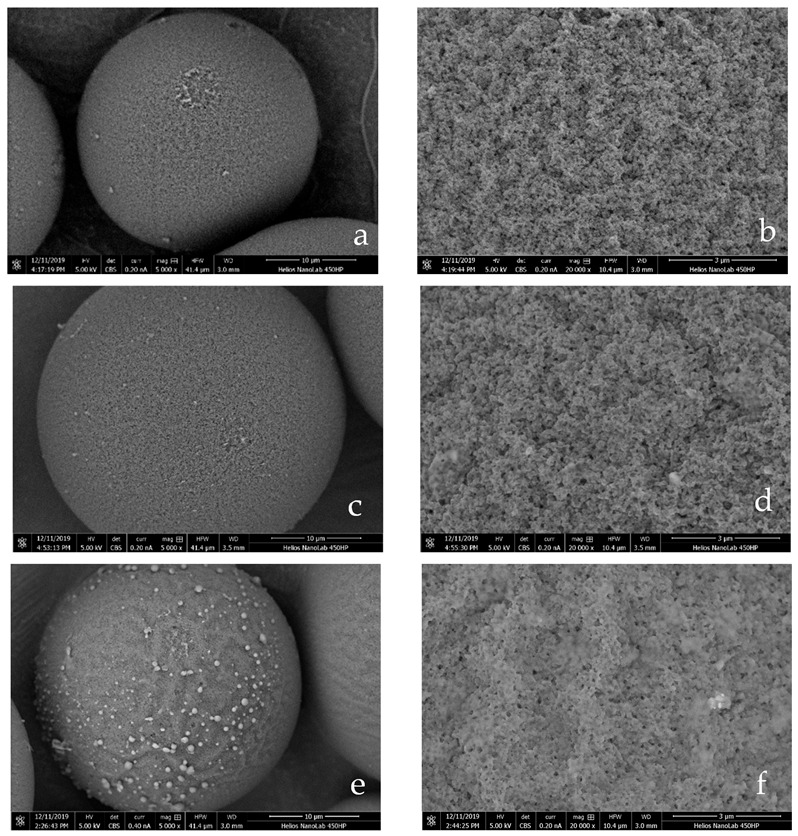
Scanning electron microscopy (SEM) micrographs of: (**a**,**b**) NIP prepared without BPA, with: (**a**) ×5000 magnification, (**b**), ×20,000 magnification; (**c**,**d**) MIP-1 prepared with 7 wt.% of BPA, with: (**c**) ×5000 magnification, (**d**) ×20,000 magnification; (**e**,**f**) MIP-2 prepared with 5 wt.% of BPA, with: (**e**) ×5000 magnification (**f**), ×20,000 magnification.

The TSMIP was prepared by the non-covalent technique of molecular imprinting. Preparation of monodisperse microspheres with a relatively small diameter is one of the important tasks for engineering materials. For this reason, it was decided to carry out the synthesis of these materials by the membrane emulsification of monomers mixtures in the first stage of the method. After this step, the obtained emulsions of monomers were polymerized next. This method of synthesis allowed one to obtain polymer beads with a regular shape (see Figure 1) and a narrow size distribution (see Table 2). Using this type of synthesis, three types of polymers were obtained. Two of the polymerizations were performed using BPA as the template, AIBN as the initiator, functional monomers (NIPAM and MMA), and a crosslinker (EGDMA). As a porogen, toluene was employed. The composition of the monomer’s ratio and the amount of crosslinker were the same as in our previous work in which thermosensitive MIPs were obtained [48]. Additionally, a non-imprinted polymer (NIP) was also synthesized to compare its properties with those of imprinted analogues. To avoid destruction of formatted BPA and functional monomer complexes, all polymerizations were performed at a temperature of about 60 °C. To determine the optimal amount of BPA, polymerizations with different template weight ratios (5 and 7 wt.%) were carried out. The BPA contents in the reaction mixture were selected based on our previous study with MIPs containing BPA templates [49]. In these materials, MIPs were obtained from the reaction mixture containing 7 wt.% BPA. Therefore, MIP-1 was synthesized at this template concentration. Unfortunately, this material was created with little efficiency, despite an increase in the amount of AIBN in the polymerization mixture. Therefore, it was decided to obtain MIP-2 from a mixture containing 5 wt.% of BPA. The amount of template in the mixture was not reduced to 3 wt.% because it was also a result of our experience with TMIPs containing diethyl phthalate imprints, where we noticed that the cavity content must be properly correlated with the thermosensitive unit content and should not be too small [48]. We noticed that when there was a too small number of imprints in the matrix, the decisive role in sorption was played by the porous matrix of the polymers itself, and not the imprints present in it. During the synthesis of MIPs, it was decided to increase the amount of the initiator up to 4 wt.%, because in the first attempts of synthesis, it was observed that at 1, 2, or 3 wt.% of AIBN contents in the reaction mixture, polymerization did not occur at all or polymerization required increasing the temperature and extending the reaction time. This was probably caused by the addition of BPA to the reaction mixture. The initiator radicals could have been caught by the molecules of the template because it contains an aromatic ring. To avoid this situation, in the next trials of MIPs polymerization, it was decided to increase the amount of AIBN to 4 wt.%. This stage of research showed that the composition of the polymerization mixture (especially the amount of BPA) was found to have a large influence on the course of the polymerization process and its efficiency. It also affected the physicochemical and sorption properties of the materials synthesized, which will be discussed in the next paragraph of this paper.

After polymerization, the size and polydispersity of the prepared polymers were investigated using DLS analysis (see Table 2). Shapes of the polymer microspheres were observed by SEM analysis. The average diameter of all microspheres was observed to be approximately 40 μm and was twice that of the pore diameter of the membrane used during the membrane emulsification process. It is generally observed that the droplet size (*d_d_*) of an emulsion can be related to the pore size (*d_p_*) of the membrane by a linear relationship (*d_d_ = xd_p_*) for given operating conditions (transmembrane pressure, dispersed phase flux, etc.), where x can range typically from 2 to 10 [4]. The same relationship was observed during the preparation of microspheres with the two-stage method in the same commercial unit offered by Micropore Ltd. [50]. The lowest average diameter of the spheres was also about two times larger than the pore size of the membrane used, and the highest was about 8 times larger than the pore size diameter. The conditions applied in our synthesis (monomers mixture flux, stirring speed, amount of dispersed phase) were selected on the basis of previous research on the process of preparation of microspheres with the use of membrane emulsification [2,41]. These conditions allowed us to obtain the smallest possible spheres with the lowest polydispersity. SPAN values for NIP and MIP-2 were 0.8 and 1, respectively. For MIP-1 this value was the highest, which means that this material was characterized with a less uniform particle size distribution [51]. The narrower size distribution was observed in the case of material without templates. This observation was also confirmed by SEM micrographs. It seems that the addition of BPA to the monomers also had an unfavorable effect on the size and polydispersity of the synthesized microspheres.

The morphologies of the synthesized copolymers are shown in Figure 1. It can be seen that the morphologies of NIP and both MIP are not very different. It can be observed that all the materials obtained have regular spherical morphologies with well-defined porous structures.

**Figure 2:**
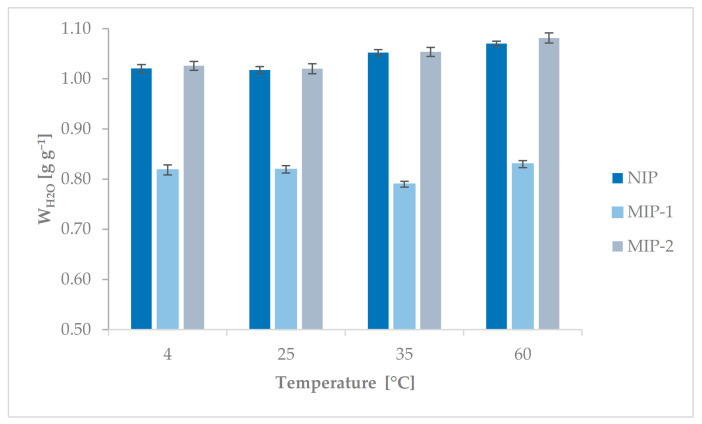
Water regain of NIP, MIP-1, and MIP-2 at different temperatures.

NIP and MIPs were examined for their contents of nitrogen and water regains (Table 2 and Figure 2). The amount of nitrogen was reduced by that derived from AIBN in both the theoretical calculation (*Z_Nteoret._*) and the determination of the actual amount of thermoresponsive components (*Z_N_*) in MIPs and NIP samples.

In the case of NIP and MIP-2, there was no difference in nitrogen content at the polymers’ surface (about 0.9 ± 0.05 mmol g^−1^). The amount of N in the MIP-1 polymer was slightly higher and was about 1.07 ± 0.05 mmol g^−1^. These values correspond to the incorporation of NIPAM into the polymer matrix with a yield of 74%, 73%, and 89% for NIP, MIP-2, and MIP-1, respectively. This meant that the thermoresponsiveness of the polymeric matrix of MIP-2 and NIP should be similar. In the case of MIP-1, the conversion of NIPAM into polymeric chains was higher and that could change the behavior of this polymer according to the change in external temperature. In earlier works [37,48], this issue was also discussed. It was noted that the number of units that respond to stimuli should not be too high because the response to external stimuli can “mask” the presence of templates in the polymeric matrix. This was observed in the case of this study as well, but this will be discussed in the section on sorption properties. However, it should be noted that the incorporation of NIPAM in the case of these materials was lower than that of our other thermoresponsive MIPs that contained a diethyl phthalate template in their structures, prepared by mass polymerization [48]. Two factors may have contributed to this unfavorable phenomenon. The first was the technique of preparation of polymers because some amount of NIPAM may be dissolved in the water phase and was not incorporated into the polymer matrix. The next one, which was mentioned before, was the presence of BPA in the polymerization mixture, which should affect the polymerization process. Free radicals could probably have been disactivated by unsubstituted groups on the aromatic rings present in the structure of this compound. The influence of the template containing aromatic rings during the imprinting process was also investigated by Maciejewska et al. [52], who noted at the beginning of their research, during the removal of the template from the polymer matrix, many peaks in the chromatogram, which they assigned to the template’s derivatives. They checked that the transformation of the template started in the pre-polymerization system. They assumed that during the preparation of MIPs, the process of free radical cascades occurs in their pre-polymerization mixture [52]. In our case, it is probable that a similar process occurs during the creation of materials, especially in the case of a too large amount of BPA in the polymerization mixture, which had a negative impact on the quality of the materials obtained. This occurrence resulted in difficulties during synthesis and, as a result, lower polymerization efficiency.

Since the prepared polymers had a thermoresponsive unit in their polymeric matrix, the water regain at different temperatures was also investigated. The thermally sensitive part of the polymer was the NIPAM mers. Poly(N-isopropylacrylamide) is the most studied thermally actuating polymer, with thermoreversible gelation properties in aqueous solutions. It gells at temperatures in the range of 32–35 °C and turns into a solution upon cooling. The value of this characteristic temperature depends on many factors, for example, while NIPAM is a part of the copolymer, it can be changed according to the composition of polymer chains [36,53] or the amount of crosslinking agent [41]. Reversibility of the hydrophilic/hydrophobic state occurs by varying the temperature below or above a critical value, well known as the lower critical solution temperature (LCST) [54]. In the case of crosslinked polymers, as a result of the change in external temperature, the prepared materials can swell below the LCST, and the water regain could be slightly higher. In the case of the synthesized MIPs and NIP, the addition of NIPAM was not too large, so the change in the *W_H2O_* value at the temperatures studied was also not so significant. There were no significant differences in the values of water regains for the given sample according to the change in temperature. As expected, the *W_H2O_* values were similar for two samples, NIP and MIP-2. In the case of MIP-1, the water regain values were lower compared to the other two polymers (MIP-2 and NIP). As mentioned before, probably in the case of MIP-1, the amount of BPA in the polymerization mixture was too high, which influenced the course of the polymerization process. Too high a concentration of BPA in the reaction mixture also had a negative effect on the quality of the cavities formed in the polymer matrix, as well as on the structure and physical and chemical properties of MIP-1. This phenomenon was also discussed by Qiu and Li et al. [55]. It was observed that increasing the amount of BPA in the reaction mixture results in the formation of more heterogeneous types of synthesized materials. This was explained by nucleation of the BPA precipitation. Bisphenol A dissolves well in monomers (in our case in EGDMA and MMA) but does not dissolve in the porous agent. However, the solubility of BPA steadily decreases as the content of toluene further increases, which is just the incident that occurred as the monomer and crosslinker were converted into copolymers through the manner of polymerization. It is likely that when too much BPA was added to the reaction mixture (in our case, it was 7 wt.%), the undissolved fine BPA particles in the oil phase played the role of a natural nucleating agent. Therefore, MIP-1 prepared with the highest amount of BPA had properties different from those of the other two polymers, MIP-1 and NIP.

The porous structures of the analyzed materials were characterized by nitrogen adsorption and desorption. This analysis provides valuable information on the morphological characteristics of the prepared sorption materials by defining the particle pore volume, the specific surface area, and the pore size distribution [56]. The porosity characterization for the analyzed sorbents is presented in Table 3. Interpreting the results obtained from the analysis of nitrogen adsorption/desorption of the developed NIP and MIP polymers gives the possibility to assess the most important polymerization reaction factors that influence the applicability of the prepared polymer material as a sorption medium and the basic factors that could affect the sorption abilities [56]. Based on the results of the BET and BJH analysis, it was observed that the differences between the investigated samples were not so significant; all revealed almost the same specific surface areas as 151, 163, and 153 m^2^ g^−1^ for NIP, MIP-1 and MIP-2, respectively. The values of the average pore sizes were very similar for NIP and MIP-2. MIP-1 was characterized with a slightly higher value of the average pore size (see Table 3). However, all materials, according to the definition of IUPAC, are mesoporous materials, as they have an average pore size in the range of 2–50 nm [57]. The values of the total pore volumes are different to some extent. The lowest is about 0.44 cm^3^ g^−1^ for NIP, while it is 0.52 cm^3^ g^−1^ for MIP-2. The highest value for MIP-1 was obtained for MIP-1 as 0.77 cm^3^ g^−1^. All values presented in Table 3 are the highest for MIP-1; this is probably due to the too high concentration of BPA in the reaction mixture (see Table 1). As mentioned before, the concentration of bisphenol A in the polymerization mixture has a significant influence on the properties of the MIPs obtained [52,55]. Probably, the precipitation of BPA from the reaction mixture during the polymerization process also resulted in the formation of a more developed surface area. Additionally, it seems that increasing the amount of AIBN in the polymerization mixture did not affect the porous structure of the polymers because, as can be seen in Table 3, the values of the average pore diameter, the BET specific surface area, and the total pore volume for MIP-2 and NIP are very similar to each other.

**Figure 3:**
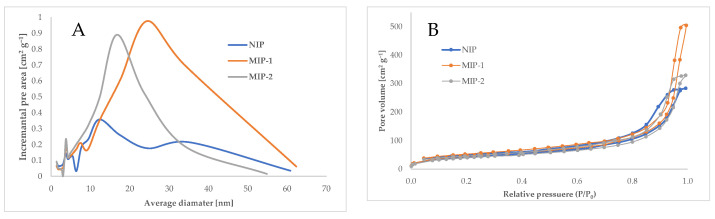
(**A**) Pore size distribution of analyzed materials; (**B**) Nitrogen adsorption isotherms for all investigated materials.

Furthermore, we compared the pore size distribution of all polymers (Figure 3A). As is well known, materials with narrow pore size distributions are very desirable as an adsorbent. This analysis showed the effect of polymerization mixtures (AIBN content and amount of BPA) on the porous structure of the synthesized materials. As can be seen, the porous structure was more ordered for MIP-2, and this polymer was characterized by a narrower mesopore size distribution, compared to NIP and MIP-1. In the case of MIP-1, the porous structure was least ordered. This unfavorable phenomenon may have been due to a too high concentration of BPA (7 wt.%) in the reaction mixture, which also had a major influence, as can be seen in Figure 3A, on ordering the structure of MIP-1, and later also on the sorption properties, which will be discussed in the next paragraph.

Moreover, the sorption isotherms were analyzed. As can be seen (see Figure 3B), all the analyzed materials revealed type IV physisorption isotherms with characteristic hysteresis loops that are related to the capillary condensation, occurring in mesopores, and with monolayer-multilayer adsorption [58,59]. This type of hysteresis is typical for mesoporous materials. The shape of the hysteresis loops is related to the specific structure of the pores. Here, the same type of hysteresis loops was observed for all synthesized polymers (NIP, MIP-1, and MIP-2), indicating the same pore structures of all materials. The revealed hysteresis loop that refers to H3 type characteristic for slit-shaped pores [58].

**Figure 4:**
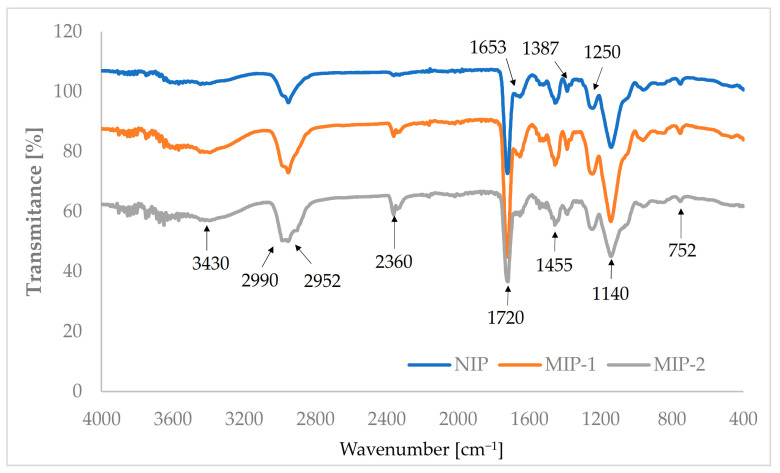
FTIR spectra recorded for NIP, MIP-1, and MIP-2.

The chemical structure of all studied polymers was investigated using the ATR-FTIR method (Figure 4). For all of these materials, their spectra are similar, and it was confirmed that the copolymerization of N-isopropylacrylamide, methyl methacrylate, and ethylene glycol dimethacrylate was completed. Figure 4 shows an absorption band from 1140 cm^−1^ and 1250 cm^−1^, which can be attributed to the C–O–C stretching vibration. A sharp intense peak at about 1720 cm^−1^ appeared due to the presence of stretching vibrations of the C=O group, and it shows the presence of the acrylate carboxyl group [60,61]. The adsorption band at 1455 cm^−1^ can be attributed to the bending vibration of the C–H bonds of the –CH_3_ group [61]. The two bands at 1387 cm^−1^ and 752 cm^−1^ can be attributed to vibrations of the α-methyl group [61,62]. The two bands at 2990 cm^−1^ and 2952 cm^−1^ can be assigned to the C–H bond stretching vibrations of the –CH_3_ and –CH_2_- groups, respectively [61,63]. Two characteristic peaks at 1653 cm^−1^ as well as 3430 cm^−1^ correspond to the vibration of the secondary amide group in the NIPAM fragment [64]. It could be observed that low-intensity peaks at about 2360 cm^−^^1^, which was slightly more intensive for both MIPs, come from the stretching vibrations of the nitrile group (C≡N) of the initiator. These peaks were more intensive for the imprinted samples because, as previously described, during the synthesis of them, a larger amount of AIBN was used.

### 3.2. Evaluation of Sorption Properties

In this work, we focus our attention on MIPs with recognition properties toward BPA, which is one of the chemicals from the group of EDCs. To confirm the creation of specific cavities designed for the target, sorption studies were performed.

#### 3.2.1. Influence of Temperature on the Adsorption of BPA

**Figure 5:**
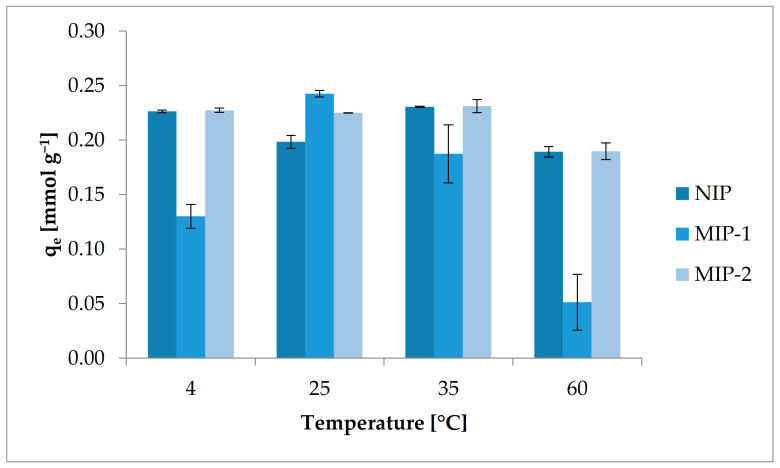
Influence of temperature on BPA adsorption by NIP, MIP-1, and MIP-2.

To study the influence of temperature on the binding of BPA by both MIPs and NIP, batch-mode sorption experiments were performed at various temperatures. The results are presented in Figure 5 and in Table S1. As mentioned before, the LCST for PNIPAM is about 32 °C, for that reason the BPA sorption evaluation was carried out at four different temperatures: (i) at 4 °C, which is much lower than the critical solution temperature, (ii) at 25 °C and 35 °C, which are close to phase transition, and (iii) at 60 °C, which is well above the critical temperature.

In the entire range of investigated temperature, BPA adsorbed on MIP-2 and NIP has the same trend. However, for MIP-1, slightly different behavior can be observed at different temperatures, and significant differences in the adsorption capacities were observed compared to NIP or MIP-2 (see Table S1 and Figure 5). The highest BPA removal efficiency was observed for MIP-1 at room temperature while for MIP-2 and NIP at 35 °C. The lowest values of adsorption for all investigated samples were observed at 60 °C. Additionally, it can be observed that the distribution coefficients reached their greatest values at a temperature well below the LCST for PNIPAM. The obtained results can be explained by two phenomena. The first is the behavior of the thermally sensitive components of the polymer matrix with a change of temperature. At a temperature below the LCST, the thermoresponsive part of the polymer matrix remains in the hydrophilic conformation, and hydrogen bonds between the water molecules and the amide groups of PNIPAM prevail here. Under these conditions, the polymer matrix is swollen, which also favors the adsorption process because there are more possible sites for the uptake of BPA from aqueous solutions. At a temperature above LCST, hydrogen bonds are broken, and the polymer is in its hydrophobic conformation, and thus the polymer matrix shrinks [54]. These conditions are not favorable to the adsorption process from aqueous solutions.

The trend of increasing BPA uptake between temperature values of 25 °C and 35 °C was maintained for MIP materials. It can be ascribed to the fact that these temperatures are close to the phase transition temperature for PNIPAM. Under these conditions, it can be supposed that probably a shape and size of cavities in MIPs had the shape and size that are close to the size and shape of the imprinted template. Moreover, not all hydrogen bonds were broken under these conditions, and the conformational transformation from a hydrophilic to a hydrophobic structure did not fully occur. Therefore, the adsorption efficiency under these conditions is influenced by two factors related to the interaction of the hydrophilic groups of the matrix with the aqueous solution, as well as the character of the imprints formed. During the sorption process from aqueous solutions at a temperature below the LCST, the sizes of cavities are larger while above this transition temperature smaller than the size of BPA molecule. The increase or decrease in the size of these characteristic cavities is probably due to swelling or shrinkage of the polymer matrix under given process conditions. This phenomenon of shrinkage or swelling can be used in the desorption and regeneration processes of polymer sorbents. The change in temperature will cause the removal of BPA from the polymer structure, for example, by loosening the polymer network and releasing the absorbed molecules.

#### 3.2.2. Adsorption Isotherms

**Figure 6:**
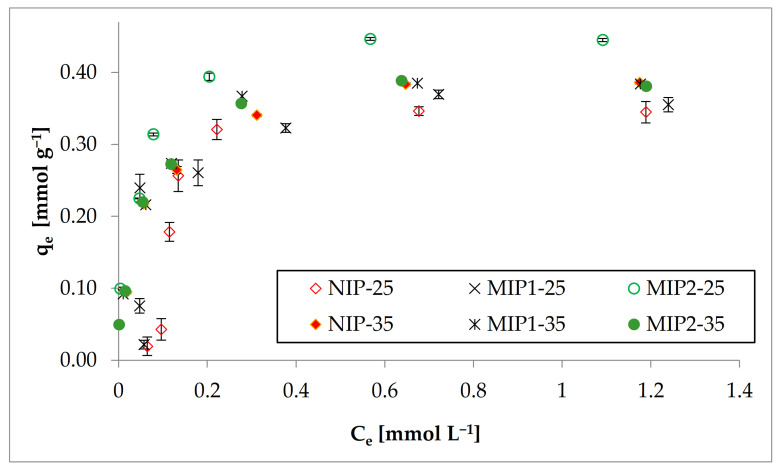
Adsorption isotherms for all studied materials at 25 °C and 35 °C.

During the evaluation of the sorption properties of the prepared sorbents, the adsorption isotherms at different temperatures were investigated (see Figure 6). It was decided to determine the isotherms at 25 °C and 35 °C because the adsorption capacities for MIPs at these temperatures were the highest. As can be observed, the adsorption behaviors of the materials were different at different temperatures. At lower temperature, all investigated polymers had similar values of the maximum adsorption capacity (about 0.35 mmol BPA g^−1^ (about 80 mg g^−1^)). Differences in BPA removal were observed at 35 °C. The highest adsorption capacity was obtained for MIP-2 at 35 °C and was about 0.45 mmol BPA g^−1^ (about 103 mg g^−1^). Moreover, as can be observed, NIP and MIP-1 at both temperatures have almost the same adsorption capacity toward BPA.

To explore the interaction between the sorbate and sorbent, sorption isotherms are used which provide a relationship between the amount of sorbate in the liquid phase at equilibrium and the sorption capacity at constant temperature. The applicability of the sorption process can be evaluated from the fundamental physiochemical data obtained by applying sorption isotherm models [65]. In recent times, linear regression analysis has been one of the most widely applied tools for defining the best-fitting adsorption models [44]. To describe the interaction of BPA with the prepared polymers, as mentioned, the three adsorption isotherm models: Langmuir, Freundlich, and Scatchard were used.

At the beginning, two typical models for the polymeric sorbent isotherm (the Langmuir model and Freundlich model) were used. All the results of these two adsorption isotherms analysis are given in Table 4.

As can be observed, MIP-2 had the most heterogeneous surface and for this material the values of the calculated $\frac{1}{n}$ parameter were the lowest (0.273 and 0.304 at 25 °C and 35 °C, respectively). For MIP-1, the respective values were slightly higher. NIP was characterized by the highest values of the characteristic heterogeneity parameter, so its surface had the least heterogeneity of all the prepared materials. This is probably related to the existence of two potential types of binding sites in the structure of MIPs, the cavities themselves formed by imprinted BPA and in the porous structure of the polymeric matrix. Whereas, for NIP, only one type of binding site existed.

Furthermore, it can be observed that the heterogeneity of the materials increases with increasing temperature. It may be related to the change of the conformation of the polymer chain from hydrophilic to more hydrophobic during the phase transition of NIPAM mers in the polymeric chains. Then, consequently, a change in the size of the pores during shrinkage of the polymeric matrix and a change in the size of the imprints also formed. When comparing the R^2^ values for the Langmuir and Freundlich models (see Table 4), the BPA adsorption data for all adsorbents agree well with the first model. The R^2^ values close to 1 were obtained by the Langmuir adsorption isotherm model; therefore, based on this model, the maximum adsorption capacities for all sorbents were calculated. According to Table 4, the highest maximum BPA adsorption capacities were obtained at 35 °C and were 0.4 mmol g^−1^ (91.3 mg g^−1^) for MIP-1 and NIP and about 0.5 mmol g^−1^ (114.1 mg g^−1^) for MIP-2. The *q_m_* values at 25 °C for all adsorbents were approximately 15% lower than the values reached at 35 °C.

To complete the sorption isotherm studies, the Scatchard approach was used. As can be seen, only for MIP-2, the Scatchard relationship showed curvature at both temperatures. The binding affinity *K^S^_BPA-_*_1_ (Table S2) for this mentioned adsorbent (MIP-2) was approximately fifty times and eight times higher as the *K_BPA-_*_2_ value of the low affinity sites at 35 °C and 25 °C, respectively. The maximum number of ligand-exchange interaction sites *N_BPA-_*_1_ was about 0.25 and 0.41 mmol g^−1^ at lower and higher temperatures, respectively. These values are almost the maximum adsorption capacities determined by the Langmuir adsorption isotherm model. It proved that most of the BPA was absorbed into the cavities dedicated to the imprinted template, and only a small part of the absorbed BPA was absorbed by the developed porous surface of the polymer. In the case of MIP-1 and NIP, the Scatchard analysis of the data fits the straight line, which is typical for a homogeneous structure with one type of binding site. Furthermore, the value of the *K^S^_BPA-2_* parameter for MIP-2, compared to the other polymers investigated, was approximately five times higher at 25 °C and slightly higher at 35 °C, also demonstrating the greater affinity of BPA for MIP-2 than for NIP and MIP-1.

These obtained materials were characterized by higher *q_m_* values than, for example, materials reported by Pereira et al. [8], for which at 35 °C the maximum sorption capacity was about 0.1 mmol g^−1^, or by Lui et al. [66], for which *q_m_* was about 0.37 mmol g^−1^, and much higher than, for example, the values reported by Cela-Pérez et al. [43] at about 30 µmol g^−1^.

#### 3.2.3. Kinetic Study

**Figure 7:**
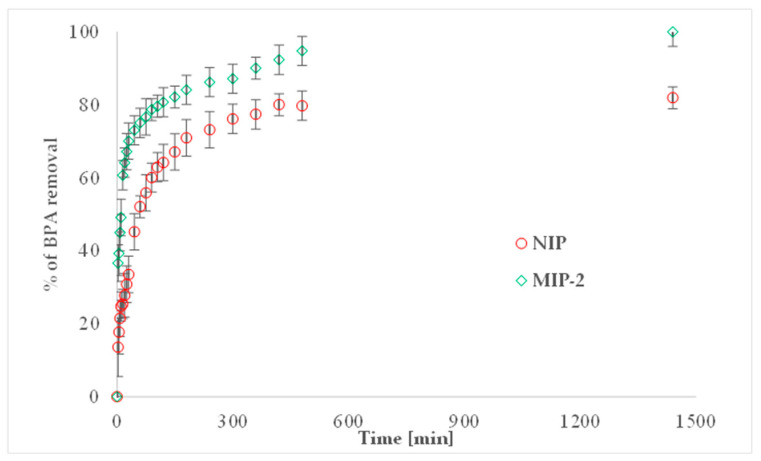
Percentage of BPA removal versus time at 35 °C for NIP and MIP-2.

Figure 7 shows the percentage of BPA removal versus time plots. The maximum BPA uptake for MIP-2 was reached within 7 h and 98% of bisphenol A removal was achieved. For NIP, the maximum uptake of BPA was reached within 8 h and the uptake was achieved at about 80%. BPA was sorbed faster at the beginning of the process and then the rate of uptake decreased. Under selected experimental conditions for the imprinted sample (MIP-2), the BPA removal process was rapid and took place during the first minutes of the study (50% of maximum uptake was observed after about 8 min). For the non-imprinted material, this process was slower—50% of maximum uptake was observed after 45 min. Earlier studies determined that the physicochemical properties of both polymers were very similar. It was observed that both were characterized by a similar amount of nitrogen, a similar specific surface area, and similar water regain values. Therefore, it could be stated that the differences in the efficiency of BPA removal between MIP-2 and NIP could be explained by the presence of additional specific sites in the MIP-2 polymeric matrix.

As mentioned, the kinetic data was fitted to the diffusion models from the second Fick law, to find what kind of diffusion (film diffusion or particle diffusion) controls the sorption process for both sorbents (MIP-2 and NIP). Table S3 shows the slope values, the linear determination coefficients, and the calculated values of *k_a_* and *k_b_*. The analysis of *k_a_* and *k_b_* can show for which resins the sorption process is faster. For MIP-2, these parameters are higher, and this means that for this sample the adsorption equilibrium was reached in a shorter time than for NIP. Additionally, analysis of the determination coefficients showed that the rate of BPA adsorption was controlled mainly by particle diffusion for both of the polymers studied.

## 4. Conclusions

The results presented confirmed that the membranes could be used for the preparation of uniform beads of MIPs. This two-stage method of synthesis allowed one to obtain monodisperse microspheres that could be used as adsorbents for BPA removal from the aqueous solutions. However, it should be emphasized that the degree of conversion of monomers in this polymerization technique is lower than that in the case of block polymerization. Nevertheless, this technique avoids the cumbersome grinding and sieving of the resultant polymers and does not destroy the resulting cavities during pretreatment. The uptake of BPA by the synthesized sorbent is mostly related to the amount of template in the reaction mixture. The amount of BPA should not be too high because it can affect the effectiveness of the formation of imprints suitable in shape and size as well as the course of the polymerization reaction. The best adsorbent (MIP-2) revealed the highest sorption capacity (0.5 mmol g^−1^) at 35 °C, and it seems that under such conditions the BPA removal process should be performed. This type of polymer can potentially be used as sensors in various analytical methods to monitor the presence of bisphenol A in various aqueous solutions and environmental samples.

## Figures and Tables

**Table 1:** Details of the synthesis conditions for each polymer.

Sample	NIPAM:MMA ^a^	M:EGDMA ^b^	BPA ^c^	AIBN ^d^	Toluene ^f^:Monomers ^e^
NIP	3:7	4:6	0	1	50:50
MIP-1	3:7	4:6	7	4	50:50
MIP-2	3:7	4:6	5	4	50:50

Abbreviations: NIPAM—N-isopropylacrylamide, MMA—methyl methacrylate, EGDMA—ethylene glycol dimethacrylate, ABIN—azoisobutyronitrile, M—functional monomers (NIPAM and MMA), **^a^** Weight ratio of functional monomers which accounted 40 wt.% of the whole weight of all monomers (functional and cross-linking), it means that in these 40 wt.%—30 wt.% is NIPAM and 70 wt.% MMA, **^b^** Weight ratio of both functional monomers (NIPAM and MMA) to crosslinking agent EGDMA, **^c^** Percentage of BPA to all monomers (functional and crosslinking), **^d^** Percentage of AIBN to all monomers (functional and crosslinking), **^e^** Weight ratio of porous agent to all total weight of all monomers (functional and crosslinking), **^f^** Porogen.

**Table 2:** Physicochemical properties of the synthesized polymers.

Sample	Average Diameter (µm)	Span	*Z_N_* (mmol g^−1^)	*Z_Nteor._* (mmol g^−1^)	Degree of Conversion [%]
NIP	38	0.8	0.90 ± 0.05	1.21	74.4
MIP-1	45	2.1	1.08 ± 0.05	1.21	89.2
MIP-2	42	1.0	0.88 ± 0.05	1.21	72.7

Abbreviations: *Z_N_*—nitrogen content, *Z_Nteor._*—theoretical nitrogen content, NIP—thermoresponsive non-imprinted polymer; MIP-1—thermoresponsive molecularly imprinted polymer, 7 wt.% of BPA; MIP-2—thermoresponsive molecularly imprinted polymer, 5 wt.% of BPA.

**Table 3:** Characterization of morphology and porous structure of the analyzed polymers.

Sample	Average Pore Diameter (nm)	BET Specific Surface Area (m^2^ g^−1^)	Total Pore Volume (cm^3^ g^−1^)
NIP	11.5	151.1 ± 2	0.41
MIP-1	19.0	163.5 ± 2	0.77
MIP-2	14.1	153.4 ± 2	0.52

Abbreviations: NIP—thermoresponsive non-imprinted polymer; MIP-1—thermoresponsive molecularly imprinted polymer, 7 wt.% of BPA; MIP-2—thermoresponsive molecularly imprinted polymer, 5 wt.% of BPA.

**Table 4:** Analysis of adsorption isotherm data for the studied samples.

Sample	T (°C)	Langmuir		Freundlich		
		*q_m_* (mmol g^−1^)	R_L_^2^	$\frac{1}{n}\;$	*A*	R_F_^2^
NIP	25	0.35 ± 0.01	0.990	0.806	0.576	0.896
MIP-1		0.36 ± 0.02	0.986	0.544	0.420	0.902
MIP-2		0.39 ± 0.01	0.999	0.273	0.516	0.933
NIP	35	0.40 ± 0.02	0.999	0.351	0.454	0.905
MIP-1		0.40 ± 0.02	0.999	0.313	0.451	0.903
MIP-2		0.48 ± 0.02	0.998	0.304	0.455	0.949

Abbreviations: NIP—thermoresponsive non-imprinted polymer; MIP-1—thermoresponsive molecularly imprinted polymer, 7 wt.% of BPA; MIP-2—thermoresponsive molecularly imprinted polymer, 5 wt.% of BPA, *q_m_*—maximal uptake of BPA, calculated from Langmuir isotherm; R_L_*^2^*—determination coefficient of linear form of Langmuir isotherm; $\frac{1}{n}$—heterogeneity index, calculated from Freundlich isotherm; R_F_^2^—determination coefficient of linear form of Freundlich isotherm; T—temperature in which the isotherms were performed.

## Data Availability

The data presented in this study are available on request from the corresponding author.

## Supplementary Materials

The following are available online at https://www.mdpi.com/article/10.3390/polym13162830/s1, Table S1: Adsorption of BPA from water solution, Table S2: Scatchard analysis, Table S3: Analysis of kinetic studies.

## Abbreviations

AIBN	Azoisobutyronitrile
ATR-FTIR	Attenuated Total Reflectance-Fourier-Transform Infrared Spectroscopy
BET	Brunauer–Emmett–Teller isotherm
BJH	Barret–Joyner–Halenda method
BPA	Bisphenol A
DLS	Dynamic Light Scattering
EDCs	Endocrine disrupting compounds
EGDMA	Ethylene glycol dimethacrylate
LCST	Lower critical solution temperature
M	Functional monomers
ME	Membrane emulsification
MIPs	Molecularly imprinted polymers
MIP-1	Thermoresponsive molecularly imprinted polymer, 7 wt.% of BPA
MIP-2	Thermoresponsive molecularly imprinted polymer, 5 wt.% of BPA
MMA	Methyl methacrylate
NIP	Thermoresponsive non-imprinted polymer, 0 wt.% of BPA
NIPAM	N-isopropylacrylamide
PNIPAM	Poly(N-isopropylacrylamide)
PSD	Pore size distribution
SEM	Scanning electron microscopy
SPAN	Characteristic polydispersity index
TSMIP	Thermoresponsive molecularly imprinted polymer
