# Supplementary material for: Membrane Emulsification Process as a Method for Obtaining Molecularly Imprinted Polymers
Source: Polymers (Basel). 2021 Aug 23;13(16):2830. doi: 10.3390/polym13162830 (PMC8400121; doi:10.3390/polym13162830)
Supplement: Supplementary file 1 [file polymers-13-02830-s001.zip › polymers-1254630-supplementary.pdf]

Table S1. Adsorption of BPA from water solution.

T	NIP	MIP-1	MIP-2
(°C)	K	K	K
4	$5.9·10^3$	$5.3·10^2$	$6.1·10^3$
25	$1.6·10^3$	$4.8·10^3$	$4.8·10^3$
35	$4.1·10^3$	$1.8·10^3$	$4.6·10^3$
60	$1.3·10^3$	$1.2·10^2$	$1.8·10^3$

Table S2. Scatchard analysis.

Sample	T (°C)	K^S_{BPA-1} (L mmol^{-1})	N_{BPA-1} (mmol g^{-1})	K^S_{BPA-2} (L mmol^{-1})
**NIP**		-	-	4.1
**MIP-1**	25	-	-	4.2
**MIP-2**		167.8	0.25	20.6
**NIP**		-	-	16.9
**MIP-1**	35	-	-	19.2
**MIP-2**		**1061.2**	0.41	20.8

Abbreviations: K^S_{BPA-1}—the first binding affinity for the first type of sites, N_{BPA-1}—number of binding sites, (the first type of sites), K^S_{BPA-2} - the first binding affinity for (the second type of sites), **NIP**—thermoresponsive non-imprinted polymer**;** **MIP-1**—thermoresponsive molecularly imprinted polymer 7 wt.% of BPA; **MIP-2**—thermoresponsive molecularly imprinted polymer, 5 wt.% of BPA.

Table S3. Analysis of kinetic studies.

Sample	$k_a t = -\ln(1 - \frac{q_t}{q_e})$		$k_b t = -\ln(1 - (\frac{q_t}{q_e})^2)$	
	k_a (min^{-1})	R^2	k_b (min^{-1})	R^2
**NIP**	$5.8·10^{-3}$	0.943	$4.6·10^{-3}$	**0.977**
**MIP-2**	$1.6·10^{-2}$	0.953	$\mathbf{1.9·10^{-2}}$	**0.993**

Abbreviations: **NIP**—thermoresponsive non-imprinted polymer**;** **MIP-2**—thermoresponsive molecularly imprinted polymer, 5 wt.% of BPA, k_a (min^{-1})-the sorption rate constant from Equation (8), k_b (min^{-1})—sorption rate constant from Equation (9).
